# Wood Anatomical Responses of European Beech to Elevation, Land Use Change, and Climate Variability in the Central Apennines, Italy

**DOI:** 10.3389/fpls.2022.855741

**Published:** 2022-03-23

**Authors:** Jose Carlos Miranda, Chiara Calderaro, Claudia Cocozza, Bruno Lasserre, Roberto Tognetti, Georg von Arx

**Affiliations:** ^1^Swiss Federal Institute for Forest, Snow and Landscape Research WSL, Birmensdorf, Switzerland; ^2^Dipartimento di Bioscienze e Territorio, Università degli Studi del Molise, Pesche, Italy; ^3^Dipartimento di Scienze e Tecnologie Agrarie, Alimentari Ambientali e Forestali, Università di Firenze, Firenze, Italy; ^4^Dipartimento di Agricoltura, Ambiente e Alimenti, Università degli Studi del Molise, Campobasso, Italy; ^5^Oeschger Centre for Climate Change Research, University of Bern, Bern, Switzerland

**Keywords:** elevational (altitudinal) gradient, *Fagus sylvatica* (European beech), hydraulic architecture, silvicultural practices, quantitative wood anatomy (QWA), xylem anatomy

## Abstract

European beech (*Fagus sylvatica* L.) is a widespread and economically important temperate tree species in Europe. The warmer temperatures and severe drought events expected in the future, especially in Mediterranean areas, could affect the vitality and productivity of beech stands that have been intensively used in these areas in the past. Here, we aim to assess the wood anatomical responses of beech to environmental variability and silvicultural practices by investigating three beech stands along an elevational gradient (1,200 to 1,950 m a.s.l.) in the Apennines (Italy). Therefore, we quantified several anatomical traits of the xylem vessels related to tree hydraulics from five trees per stand and investigated variability between and within tree rings. Our results suggest generally limited trait plasticity, with higher plasticity of mean vessel lumen area and theoretical hydraulic conductivity, while maximum vessel size and mean hydraulic diameter were less plastic, likely because of the stronger determination by tree height. High-elevation trees were hydraulically more limited than trees at a mid and lower elevation as indicated by the more conservative anatomical configuration, i.e., comparatively smaller vessels and a 50% tighter trait coordination. Cessation of coppicing resulted in a hydraulically safer anatomy with comparatively smaller vessels at the most intensively used site (1,200 m), triggered by increased water demand due to an increase in canopy density, and thus, an increase in stand transpiration. Furthermore, maximum vessel size at the beginning showed different climate sensitivity compared to the rest of the tree ring, while intra-ring anatomical profiles showed little difference between normal and the 5 years with the highest and lowest mean temperature and precipitation. Overall, this study highlights the challenges to separate the externally induced medium- to longer-term responses from ontogenetically determined patterns. We, therefore, call for more comprehensive studies to further explore and verify the plasticity of wood anatomical traits in European beech in response to short- to long-term environmental fluctuations to gain a mechanistic understanding useful for sustainable forest ecosystems.

## Introduction

European beech (*Fagus sylvatica* L.) is one of the most important forest species in Europe, in terms of nature conservation as well as exploitation (Ellenberg and Leuschner, 2010). However, climate change scenarios project that forest areas suitable for beech will decrease across Europe over the next century (Sabate et al., 2002; Gessler et al., 2007; Zimmermann et al., 2015). Physiological performance and growth of beech are thought to be adversely affected by the changing environmental conditions because of its higher sensitivity toward low water availability and longer drought periods than other temperature broad-leaved species (Ellenberg, 1996; Fotelli et al., 2002; Peuke et al., 2002; Walthert et al., 2021). Specifically, the current warming trend may accelerate the cambial growth of beech at its latitudinal and elevational limits. High summer temperatures promote the radial growth of beech, suggesting a positive effect of increasing mean summer temperature at higher latitudes or elevations on the cambial activity of beech (Bosela et al., 2018). Conversely, southern European beech populations are increasingly suffering from summer drought (Piovesan et al., 2008; Linares and Camarero, 2012), which may trigger episodes of increased tree mortality (Bolte et al., 2016; Cocozza et al., 2016). However, also in central areas of beech distribution extremely warm summers have recently been shown to damage beech trees (Schuldt et al., 2020; Walthert et al., 2021).

Beech can adjust stem hydraulic performance mainly by changing xylem vessel number, vessel diameter, vessel density, vessel connectivity, and tree growth rate (Carlquist, 2012; Giagli et al., 2016; Hacke et al., 2017). Although vessel traits are largely determined by genetics and biophysical constraints (Carlquist, 2003; Wortemann et al., 2011; Eilmann et al., 2014; Olson et al., 2014), xylem formation varies between sites and years and is closely related to changes in weather conditions, particularly water availability and air temperature (Arnic et al., 2021). In Alpine environments, secondary growth (e.g., cambium differentiation and xylem formation) of beech has been found to vary with temperature (Prislan et al., 2013) and specifically to depend mainly on June–July temperature and precipitation (Di Filippo et al., 2007). Besides, in the context of precipitation regime variation, wood anatomical trade-offs between water transport capacity and relative drought resistance may shape the performance of beech populations, as has been observed in other species (Hoeber et al., 2014; Chenlemuge et al., 2015; Kotowska et al., 2015). This trade-off occurs as hydraulic efficiency increases with vessel lumen diameter to the fourth power (Tyree and Ewers, 1991), while in contrast, the wider the vessels, the lower their hydraulic safety against frost- and drought-induced embolism (Hacke and Sperry, 2001). However, trees could also vary spatial vessel arrangement in addition to vessel dimensions to adjust overall hydraulic efficiency and safety as derived from the xylem structure. Nevertheless, the large trait plasticity of beech trees after disturbance could allow this species to withstand decreasing water availability to a certain degree (Kahle, 2006; van der Werf et al., 2007).

The Italian mountain systems provide an excellent framework in which wood anatomical differences at inter- and intra-annual levels can be expected in response to changing climatic conditions, specifically soil water supply and atmospheric evaporative demand (Tognetti et al., 2019). In the Apennines, beech usually grows above 900–1,000 m a.s.l. and is widespread on northern slopes and where precipitation and fog maintain moister air conditions (Nocentini, 2009). This lower distribution limit is shifted upward on the sunnier and warmer southern slopes. Besides, tree-ring analyses have shown distinct growth responses of beech to bioclimatical and elevational gradients (Di Filippo et al., 2012; Chiesi et al., 2013; Tognetti et al., 2014).

Forest management practices have significantly modified the distribution, composition, and structure of beech forests in the Apennines (Nocentini, 2009), where it is the most widespread forest species (Scarascia-Mugnozza et al., 2000). However, after World War II, the progressive depopulation of mountain areas due to socioeconomic changes led to the abandonment of coppice-with-standards used for the production of firewood and charcoal (Hahn and Fanta, 2001; Brunet et al., 2010). Transition or conversion from management with frequent harvesting that promotes shoot regrowth from stumps (coppice) to a naturalized forest with long-harvest intervals or even cessation of active management that promotes tree regeneration from seeds (high forest) can be performed in different ways (Salomon et al., 2017; Unrau et al., 2018). Conversion of beech coppice to high forest affects different competition-related stand traits such as stand density, stand basal area, and canopy cover, which leads to changes in stand structure and local environmental conditions, in particular soil moisture and temperature (Aussenac, 2000; Salomon et al., 2016; Zellweger et al., 2020). The impact of these changes on the underlying wood anatomical structure, and the implications for tree growth and functioning in light of the increasing climate variability is largely unknown.

The aim of this study was to investigate the intra- and inter-annual variability of wood anatomical traits of beech stands in response to an elevational gradient, the cessation of the silvicultural practices (coppicing), and years of extreme climate in the Majella National Park (Central Italy). We hypothesized to observe (i) higher plasticity in intra-annual anatomical trait responses at lower elevations compared with the higher elevations because of the expected wetter and warmer conditions (Martínez del Castillo et al., 2016) and a hydraulically more efficient anatomical structure; (ii) an increase of theoretical hydraulic safety against embolism after coppice cessation due to increased stand evapotranspiration and competition for water; and (iii) an increase in the theoretical hydraulic safety against embolism in years of extreme climate to cope with severe droughts.

## Materials and Methods

### Study Area and Land Use Changes

**FIGURE 1:**
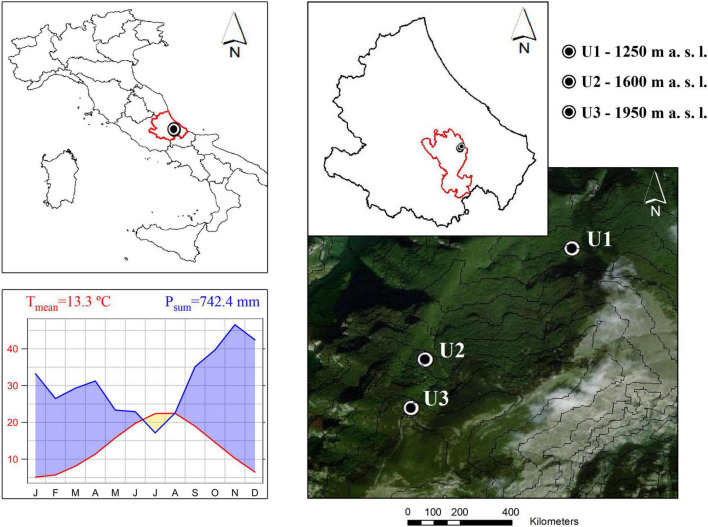
Location of the study sites and climate diagram of the study area. T_mean_—mean annual temperature, P_sum_—mean annual precipitation (CRU TS4.04 Dataset, Harris et al., 2014).

This study was conducted in the Majella National Park on European beech (*Fagus sylvatica* L.) trees. The Majella massif is a north-south ridge at 32.5 km from the Adriatic Sea, in Abruzzo (Central Italy). Three sites were selected along an elevational gradient at the north-facing slope of Mount Ugni: U1 at 1,200 m a.s.l. (42° 8′ 27″ N, 14° 10′ 24″ E), U2 at 1,600 m a.s.l. (42° 7′ 53″ N, 14° 9′ 40″ E), and U3 at 1,950 m a.s.l. (42° 7′ 38″ N, 14° 9′ 35″ E). The two lower sites, U1 and U2, are characterized by essentially pure beech stands, whereas the highest site, U3, represents the elevational limit for beech in the study region (Table 1). U3 is characterized by a transition between beech and mountain pine (*Pinus mugo* Turra spp. *mugo*), with the latter species accounting for less than 14% of all the trees and less than 10% of basal area. All the three studied sites represent stands with different age structures. The whole study area has a typical Mediterranean climate, with a mean annual temperature of 13.3°C and an annual precipitation sum of 742.4 mm during the study period (1968–2004, Harris et al., 2014). Precipitation is unevenly distributed throughout the year, with a drought period during the summer months (Figure 1). During the study period (1968–2004), the use of the forest was changed from coppicing with standards to the cessation of all the harvesting activities when Mount Ugni was declared a Natural State Reserve in 1981. It was expected that the impact of this changed forest use would decrease in the order U1 > U2 > U3, as lower elevation sites would be subject to greater harvesting pressure due to greater accessibility. This is reflected in the higher stem/stump ratio of site U1 compared with U2 and U3 (Table 1) which indirectly reflects the intensity of past land use at each site. In fact, orthophotos of U3 showed relatively constant land cover between 1954 and 2017 compared with other similar elevations in the region (Calderaro et al., 2020).

**TABLE 1:** Stand structural traits at each study site, U1–1,200 m a.s.l., U2–1,600 m a.s.l., U3–1,950 m a.s.l. BA–basal area, DBH–diameter at breast height. Where applicable, values are means ± SD.

	U1	U2	U3
No. stems	221	126	150
No. stumps	81	93	144
No. stems⋅ha^–1^	1758.7	1002.7	1193.7
No. stumps⋅ha^–1^	644.6	740.1	1145.9
No. stems⋅stump^–1^	2.8	1.4	1.0
BA (m^2^⋅ha^–1^)	31.9	28.4	27.3
DBH (cm)	12.4 ± 9.2	18.8 ± 9.5	16.5 ± 5.7
Mean tree height (m)	NA	22.8	14.7
TRW (μm)	1072.5 ± 512.5	1488.5 ± 497.6	1310.5 ± 382.7
Age of cored stems (years)	92.5 ± 21.9	85.3 ± 24.6	84.3 ± 11.7
Period with sample size *n* = 5 (years)	1950–2004	1968–2012	1946–2012

### Tree Sample Collection and Processing

**FIGURE 2:**
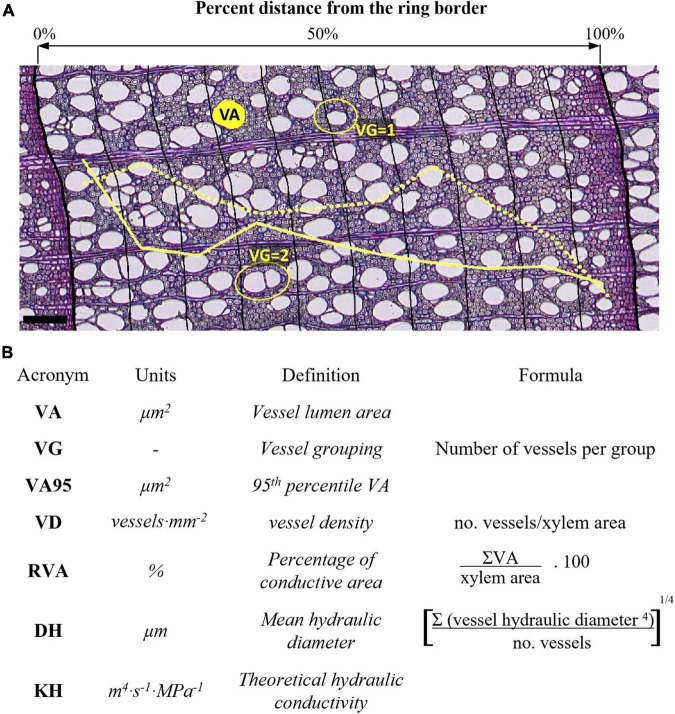
Illustration of **(A)** anatomical measurements and **(B)** anatomical traits with acronyms and formulas. Thick black lines in **(A)** denote ring borders and thin lines denote intra-ring sector limits. The solid yellow curve in **(A)** shows the relative intra-ring changes in vessel grouping (VG), the dotted yellow curve shows the relative intra-ring changes in mean vessel area (VA). Scale bar = 100 μm.

From May 2013 to September 2014, two increment cores were taken at 120° from 5 dominant trees per site with a 5-mm increment borer (Haglöf, Mora, Sweden). Tree cores were taken at breast height avoiding eccentricity and tension wood because of the slope. Subsequent vessel anatomical measurements were conducted following the procedure detailed in von Arx et al. (2016). Core samples were split into 4–5 cm long pieces and heartwood was boiled in a water-glycerin solution. Microsections of 15–20 μm thickness were cut with a rotary microtome (Leica, Heidelberg, Germany), stained with safranin (1% in distilled water), Astra blue (0.5% in distilled water and acetic acid), and fixed on permanent slides with EUKITT (BiOptica, Milan, Italy). Overlapping high-resolution digital images (0.945 pixels⋅μm^–1^) were captured with a digital camera (Canon EOS 650D, Canon Incorporation, Tokyo, Japan) connected to an Olympus BX41 microscope (Olympus Corporation, Tokyo, Japan). The images from the same microsection were stitched together with PTGui (New House Internet Services BV, Rotterdam, Netherlands). Then, images were analyzed with ROXAS version 3.0.578 (von Arx and Carrer, 2014), which depends on Image-Pro Plus version 6.1 or higher (Media Cybernetics, Silver Spring, MD, United States). Image analysis covered a common period from 1968 to 2004 that was represented by *n* = 5 trees at all the sites and produced the following individual vessel data (Figure 2): vessel lumen area (VA), vessel grouping index (VG; von Arx et al., 2013), and theoretical hydraulic conductivity (KH) according to the Hagen–Poiseuille law (Tyree and Zimmermann, 2002). Further vessel traits were calculated at intra-ring sector or full ring level: number of vessels and analyzed xylem area, 95th percentile of vessel lumen area (VA95), vessel density as the number of vessels per analyzed xylem area (VD), proportion of accumulated vessel lumen to xylem area or relative vessel area (RVA), and mean hydraulic diameter (DH; Tyree and Zimmermann (2002). VA, VA95, DH, and KH features are all related different aspects of theoretical hydraulic efficiency with the first three showing mean properties while KH reflects accumulative water transport capacity. We distinguish between VA95 and VA because the widest vessels reflect maximum transport efficiency but are putatively most vulnerable to embolism (Tyree and Ewers, 1991; von Arx et al., 2012), although variation in vessel size is primarily a function of tree height (Carrer et al., 2015; Rosell et al., 2017). VG, VD, and RVA characterize the spatial vessel arrangement and integration, which affects hydraulic efficiency and safety (Carlquist, 2003; Nardini et al., 2012). These traits reflect different components of the theoretical hydraulic safety and efficiency of xylem vessels, but also of the entire xylem including mostly mechanically and physiologically important fiber and parenchyma cells (Bittencourt et al., 2016; Lourenço et al., 2022).

### Climate Data and Selection of Extreme Years

Monthly mean temperature and precipitation sums of the area were derived from the CRU TS 4.04 gridded dataset with 0.5° spatial resolution (Harris et al., 2014). These data were used to identify significant correlations with anatomical traits of the ring sectors. They were used as well to identify years with extreme climate. For this purpose, we selected the 5 years with the highest and lowest mean temperature and precipitation values of the June to August period of the series as “extreme years” (Jetschke et al., 2019). The period of June, July, and August was selected because seasonal drought occurs during this period and to allow comparison with previous studies in the same area and in the Mediterranean Basin (Di Filippo et al., 2007; Calderaro et al., 2020) that showed correlations between anatomical traits and/or tree ring widths and climate conditions during these months. The selected extreme years with high and low temperatures and precipitations were above and below the 85th and 15th percentile of the study period and also of all the available climate series (1901–2016), respectively.

### Data Standardization and Statistical Analysis

In order to evaluate the modification of wood anatomical responses to different elevation, climate variability, and land uses, we examined vessel traits at the intra-ring level. To that end, vessels were assigned to one of 5 or 10 tangential sectors of equal width based on their position in the ring (Figure 2; Sass and Eckstein, 1995). The higher sectorization (10) was performed in order to obtain a semicontinuous intra-ring profile of anatomical traits with most of the sectors fulfilling the following criteria: sample depth consisting of five trees per site (study period 1968–2004), sectors containing a minimum of five vessels (0.66% of the total number of sectors did not meet this criterion and were discarded), and sectors being wider than the maximum vessel radius of the sector itself (0.5% of the rings did not fulfill this criterion). To compare wood formed early and late in the season, we refer to the early formed wood (E_f_W) as the wood that is at the relative position of 0–50% in the ring, and to the late-formed wood (L_f_W) as the wood that is at the relative position 50–100% in the ring. The lower sectorization (5) was performed to get robust sector estimates based on the aforementioned criteria, the number of which additionally roughly corresponded to the estimated number of months of wood formation.

As vessel dimensions have an increasing trend during ontogeny driven by distance from the distal tree parts (Supplementary Figure 1; Carrer et al., 2015), two standardizations methods were performed:

(i) indexed values (X_ind_) were used to assess the correlations in anatomical traits among each other at the ring and intra-ring level and with monthly climate data. Therefore, for each core and anatomical trait, a time series was built per intra-ring sector, representing inter-annual variations of the respective trait at the intra-ring level. Then, we fitted a cubic smoothing spline with 50% frequency cutoff at 30 years to each anatomical series to remove low-frequency trends introduced by tree height growth, land use changes, and other disturbances [using dplR package in R; Bunn (2008)]. The detrended index was calculated as the ratio between the observed and the fitted values for each ring (Cook and Kairiukstis, 1990). Monthly climate data were detrended in the same way, to avoid biased correlations with anatomical features because of possible trends in climatic data (Klesse, 2021).

(ii) Standardized values (X_std_) were used to compare the differences between intra-annual patterns at the different sites, land use regimes, and climatic extreme conditions. Standardization consisted of dividing individual intra-annual sector values by the mean annual value of the corresponding ring in each tree. With this method, we standardized using only same year data in the different sectors, thus, avoiding bias through trends and range shifts among years.

To test for anatomical differences among sites, land use intensities, and annual climate extremes, a permutation test of independence was applied as data did not meet normality assumptions. This test allows comparing the distribution of the values, which is of central interest in the case of anatomical values at the ring and sector level. Further pairwise permutation *t*-tests (*post-hoc* tests) were performed to compare between sites, periods, and extreme vs. normal climate years.

The general statistical robustness of each anatomical chronology was assessed by the average correlation between series (Rbar) and the expressed population signal (EPS). The first statistics is an estimate of the percent common variance between series, while the second statistics provides an estimate of how well the selected series reflect the entire population (Wigley et al., 1984). In general, anatomical chronologies showed weak robustness (Table 2) except for VA95 and TRW (Rbar = 0.32 to 0.61, and EPS = 0.68 to 0.89). Consequently, monthly climate correlation analyses were only performed for these traits to reduce the likelihood of spurious correlations. Spearman’s correlation coefficients were calculated between indexed monthly temperature and precipitation data and each sector chronology. Correlations were performed with indexed monthly climatic values for the previous and current growing seasons to allow for lagged and immediate associations.

**TABLE 2:** Descriptive statistics of the different wood anatomical chronologies at each site. Rbar–mean inter-series correlation; EPS–expressed population signal. Period 1968–2004 (*n* = 5). For anatomical trait abbreviations, see Figure 2.

Trait	Site	Rbar	EPS
VA	U1	0.12	0.38
	U2	0.27	0.65
	U3	0.10	0.36
VA95	U1	0.32	0.68
	U2	0.45	0.80
	U3	0.41	0.78
DH	U1	0.32	0.68
	U2	0.31	0.69
	U3	0.33	0.71
KH	U1	0.05	0.20
	U2	0.23	0.60
	U3	0.12	0.41
VD	U1	0.16	0.46
	U2	0.14	0.44
	U3	0.22	0.59
RVA	U1	0.08	0.28
	U2	0.41	0.78
	U3	0.20	0.56
VG	U1	0.35	0.71
	U2	0.15	0.46
	U3	0.14	0.46
TRW	U1	0.47	0.81
	U2	0.61	0.89
	U3	0.38	0.76
TRW*	U1	0.48	0.88
	U2	0.44	0.91
	U3	0.49	0.92

*Data derived from a more comprehensive dendrochronological analysis with 15 individuals including the study trees.

*P*-values were corrected in all analyses following the Benjamini-Hochberg method to avoid inflation of the type I error. A significance level α = 5% was considered for all the performed analyses. The study period covered from 1968 to 2004, with additional focus on the periods before the land use change (1968–1981) and the period 10 years after the harvesting was ceased (1990–2004). Statistical analyses were performed using R version 4.0.3 (R Core Team, 2019) and the packages coin (Hothorn et al., 2008), rcompanion (Mangiafico, 2020), nlme (Pinheiro et al., 2020), and dplR (Bunn, 2008). Figures were produced using the package ggplot2 (Wickham, 2009).

## Results

### Absolute and Intra-Annual Differences Related to the Site and Land Use Change

**FIGURE 3:**
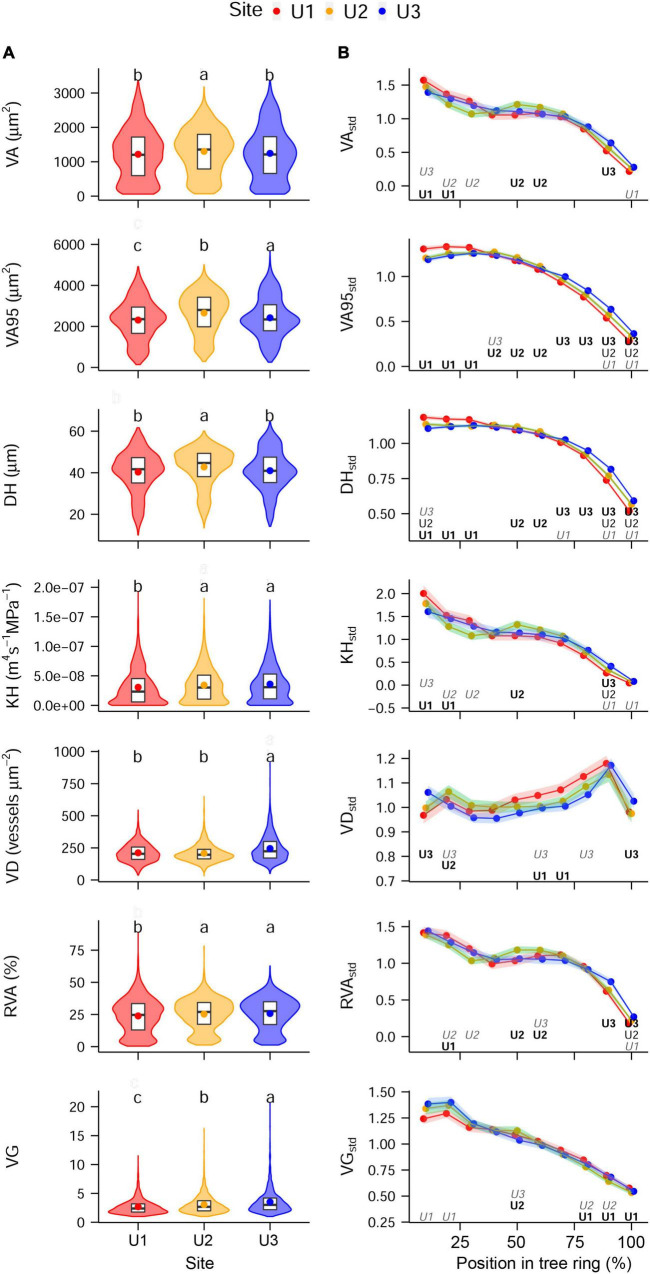
**(A)** Absolute inter-annual and **(B)** standardized intra-annual values of each of the anatomical traits studied at the different study sites: U1—1,200 m a.s.l., U2—1,600 m a.s.l., and U3—1,950 m a.s.l. Panel **(A)** shows violin plots, with boxplots (1st, 2nd, and 3rd quartiles) and mean values (dots); letters indicate significant differences between sites (permutation test of independence, *P*-value < 0.05). Panel **(B)** shows mean values (dots) and 95% CI (shaded area); letters indicate if values are significantly higher (**bold**), lower (*italics gray*), or if they are significantly different at all sites (regular format indicates the middle value). If both **bold** and *italics gray* are shown, this means that the mid-value was the only one that did not differ significantly. Permutation test of independence, *P*-value < 0.05. For the anatomical trait abbreviations, see Figure 2.

Overall, absolute mean anatomical traits and standardized intra-ring profiles proved to be remarkably similar among sites (Figure 3A). On average, vessel area consisted of about one-fourth of the total area of each sector ranging from 0.4 to 89.1%. Within the observed differences in distributions of the absolute values (Figure 3A), U2 showed the highest median of VA, VA95, and DH, compared with the other sites, whereas U3 showed the highest median in VD and VG. VA95, KH, RVA, and VG showed lower medians at U1 compared with the other sites. Absolute TRW values showed no significant differences among sites during the study period. The standardized values of all the anatomical traits, except for VD_std_, showed the following general intra-ring pattern: stable to slightly decreasing values between the beginning and the relative position 50% in the ring and a clear decreasing trend from there to the end of the ring (Figure 3B). In contrast, VD_std_ peaked at the relative position of 90% in the ring independent from the site. Despite the generally similar intra-ring profiles, anatomical traits showed significant fine-scale differences among sites (Figure 3B). Particularly, intra-ring profiles of VA95_std_ and DH_std_ differed significantly among sites in almost all sectors, where U1, U2, and U3 presented the highest values in the first (0–30%), second (40–60%), and third (70–100%) relative positions of the ring, respectively.

**FIGURE 4:**
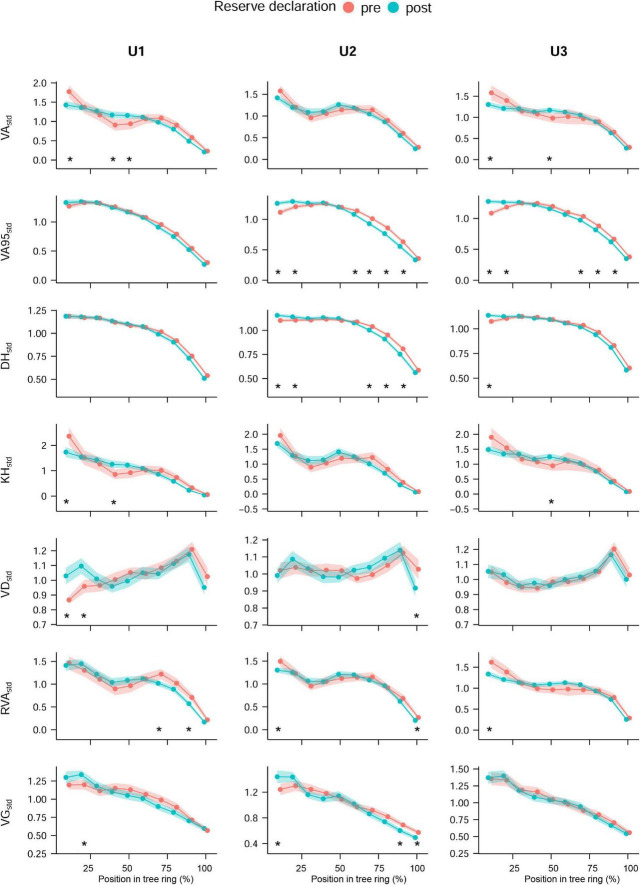
Standardized intra-annual values of each land use period. Land use periods refer to the periods before (1968–1981) and after (1990–2004) the declaration of Ugni as Natural State Reserve in 1981 at the different study sites, which coincided with the cessation of coppicing: U1—1,200 m a.s.l.; U2—1,600 m a.s.l.; U3—1,950 m a.s.l. Mean values (dots) and 95% CI (shaded area); asterisks indicate significant differences in sector values. Permutation test of independence, *P*-value < 0.05. For the anatomical trait abbreviations, see Figure 2.

There were also differences in anatomical traits between the periods before (1968–1981) and after (1990–2004) the land use change, when Ugni was declared a Natural State Reserve and competition increased generally, with the largest impact at the lowest site (U1) and the smallest impact at the highest site (U3) (Figure 4 and Supplementary Figures 2, 3). Absolute trait values were generally higher in the period after the forest land use change, but there were inversed relationships for VD, TRW (both at all sites), and VG (at U1) and no significant differences for VA95 (all the sites) and DH (at U3). Conspicuous differences between sites in intra-annual patterns were visible in VA95*_*std*_* (U2 and U3) and DH (at U2) with significantly higher values in the first part of the ring (relative position 10 and 20%) in the period after the land use change and lower almost at the end of the growing season (relative positions 70, 80, and 90%).

### Correlation Among Anatomical Traits at the Ring and Intra-Ring Level

**FIGURE 5:**
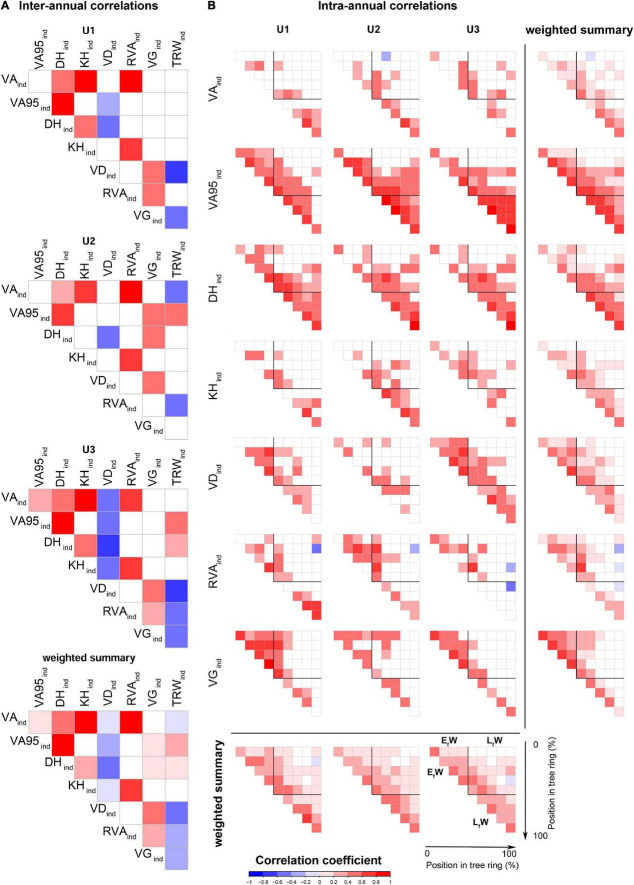
**(A)** Inter-annual Spearman’s correlations among all indexed series of anatomical traits and **(B)** intra-annual Spearman’s correlations in each of the sector’s indexed series of anatomical traits at the three study sites: U1—1,200 m a.s.l.; U2—1,600 m a.s.l.; U3—1,950 m a.s.l. Colors represent the correlation coefficient values (r). Only significant correlations (*P*-value < 0.05) are presented. Weighted summarized values are calculated as the sum of the significant correlation coefficients of the sites and traits, divided by the total number of sites and traits, respectively. E_f_W: Early formed wood (0–50% of the ring); L_f_W: late formed wood (50–100%), assuming each corresponds to half of each ring. For the anatomical trait abbreviations, see Figure 2.

The correlation analyses between standardized ring-level values revealed many associations among anatomical traits and ring width, particularly at U3 (Figure 5A). The triplet formed by VA*_*ind*_*, KH*_*ind*_*, and RVA*_*ind*_*, strongly correlated with each other at all sites as it was also the case between DH*_*ind*_* and VA95*_*ind*_*. Site U1 showed the lowest number of correlations between anatomical traits and TRW*_*ind*_* (only VD*_*ind*_* and VG*_*ind*_*). At all the sites, significant correlations were generally positive aside from the ones of VD*_*ind*_* that were all negative except for its positive correlation with VG*_*ind*_*, and the generally negative correlations of TRW*_*ind*_* with all the other traits except VA95*_*ind*_* and DH*_*ind*_*.

Anatomical trait correlations at the intra-ring level showed different patterns of each trait across tree rings and also differences among sites (Figure 5B). In general, anatomical traits from nearby parts of the ring and thus formed at similar times of the growing season were positively correlated. VA95*_*ind*_* showed the strongest correlations between sectors throughout the ring followed by DH*_*ind*_*. DH*_*ind*_* values correlated significantly in 90% or more of all pairs of the L_*f*_W sectors at all sites. VA95*_*ind*_* showed a similar pattern in L_*f*_W except for the last formed sector in U1 trees. In contrast, VA*_*ind*_* and KH*_*ind*_* showed few significant correlations between the different sectors at all sites, with KH*_*ind*_* showing more significant correlations in L_*f*_W sectors at U2.

### Climate Correlations and Anatomical Trait Variation in Years With Extreme Climate

**FIGURE 6:**
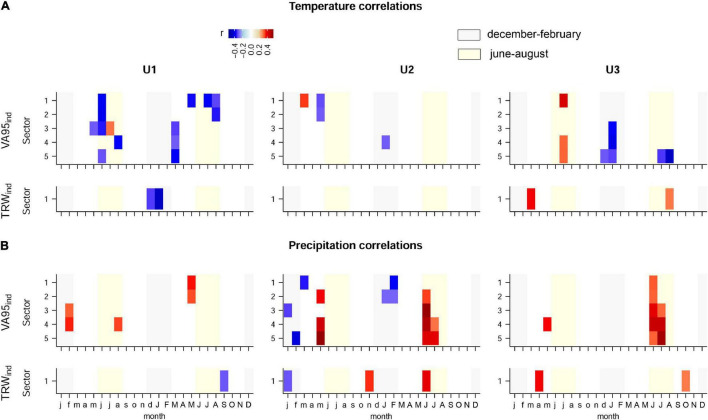
The Spearmans’ correlation of the tree-ring sector indexed series of VA95, DH, and TRW with monthly **(A)** temperature and **(B)** precipitation at the three study sites: U1—1,200 m a.s.l.; U2—1,600 m a.s.l.; U3—1,950 m a.s.l. As an annual value, TRW has not been divided into five sectors. Colors represent the correlation coefficient values (r). Only significant correlations (*P*-value < 0.05) are presented. Lower case letters indicate the initials of the previous-year’s months, while capital letters indicate the initials of the current year’s months. For the anatomical trait abbreviations, see Figure 2.

As specified before, climate correlation analyses were performed only for VA95 and TRW traits. Other trait chronologies showed moderate (DH), low (RVA, VG, and VD) to very low (VA, KH) robustness (Table 2). Significant results included general negative associations of VA95*_*ind*_* sectors with previous summer (June), and current March and summer temperatures (July–August) at U1, and positive and negative associations with the previous summer (July) and winter (December–January) temperatures, respectively, at U3 (Figure 6A). Furthermore, positive associations of VA95*_*ind*_* with current summer precipitations (June–July) were observed at U2 and U3, and negative associations with winter precipitation (January–February) at U2 (Figure 6B). These correlation patterns were largely different from those of TRW_ind_, which showed a negative correlation with temperature in the previous winter (December–January) at U1.

**FIGURE 7:**
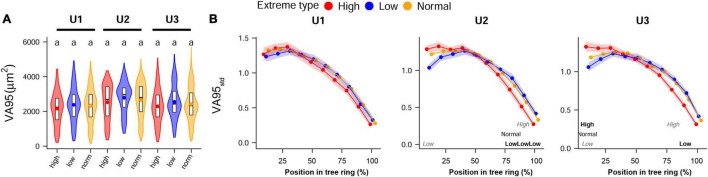
**(A)** Inter-annual and **(B)** standardized intra-annual values (standardized to the annual mean) of VA95 studied at the 5 years with the highest and lowest temperatures during the dry trimester June–July–August in the time period 1968–2004 at the different study sites: U1—1,200 m a.s.l.; U2—1,600 m a.s.l.; U3—1,950 m a.s.l. Panel **(A)** shows violin plots, with boxplots (1st, 2nd, and 3rd quartiles), and mean values (dots); letters indicate significant differences between sites. Panel **(B)** shows mean values (dots) and 95% CI (shaded area); letters indicate if values are significantly higher (**bold**), lower (*italics gray*), or if they are significantly different at all sites (regular format indicates the middle value). If both the **bold** and *italics gray* are shown, this means that the mid-value was the only one that did not differ significantly. Permutation test of independence, *P* < 0.05. For the anatomical trait abbreviations, Figure 2.

Analysis of anatomical traits in years with extreme climate showed scarce differences between years with extreme high and low temperatures and also between them and average years (Figure 7). Inter-annual patterns were fairly similar among years with extremely high and normal temperatures, but lower temperature years slightly affected the first and last sectors of VA95*_*std*_* at U2 and U3 sites. No significant differences between normal and extreme summer precipitation years were observed in intra-annual patterns (data not shown). Both the TRW absolute values and TRW_ind_ showed no significant differences between extreme high, low, and normal temperature and precipitation years (Supplementary Figure 2).

## Discussion

### The Anatomical Structure Shows Little Trait Plasticity Apart From Species-Specific Patterns

Different anatomical traits showed different levels of plasticity in response to environmental conditions and land use regime at each site as observed in the previous studies in beech (Fonti and Jansen, 2012; Oladi et al., 2014; Diaconu et al., 2016). The general trend of higher to mid and lower values of all the studied traits (except for VD*_*std*_*) across the tree rings reflects the species-specific wood anatomy of beech, which is characteristic for a diffuse-porous to semi-ring-porous species (Bosshard, 1982; Schweingruber, 2007). The observed wider vessels and the higher vessel grouping in the earlywood are consistent with maximizing hydraulic efficiency in this part of the ring. This fits the higher water availability and lower evaporative demand from the canopy at the beginning compared to the end of the growing season (Sass and Eckstein, 1995; Prislan et al., 2018). Similarly, the increasing trend of VD*_*std*_* toward the end of the growing season, peaking at relative positions 70 and 80% compensates for the hydraulically less efficient smaller, but putatively safer vessels produced in the latewood (lower VA*_*std*_* and VA95*_*std*_*).

Apart from these general and mostly species-specific patterns in wood anatomical traits, strongly correlated maximum vessel size and hydraulic diameters across tree-rings with narrow confidence bands (Figures 3, 5) suggests that these beech stands poorly regulate hydraulic responses to changes in water availability throughout the year. The low plasticity of VA95 and DH is likely related to the biophysical constraints associated with tree height (Olson et al., 2014; Carrer et al., 2015). In contrast, the strongly correlated trait group of VA, KH, and RVA showed higher intra-annual plasticity, which has also been observed in previous studies in responses to environmental fluctuations (Oladi et al., 2014; Diaconu et al., 2016). Xylem trait plasticity could be expected considering the wide distribution range of the species, both globally (longitudinally and altitudinally) and locally (elevational band in the Apennines). In fact, beech is known to have high-genetic diversity within populations, resulting in phenotypic plasticity, i.e., short-term acclimation (Bresson et al., 2011; Wortemann et al., 2011; Cocozza et al., 2016). However, the intra-annual plasticity of VA, KH, and RVA could be at least partly related to the less robust data for these traits according to Rbar and EPS statistics (cf. Table 2), suggesting the limited number of replicates taken per site (*n* = 5) only allow identifying the strongest relationships.

### Plasticity Decreases and Hydraulic Safety Increases Toward the Highest Site

Results supported our first hypothesis (i): trees had less plastic and a hydraulically safer anatomical structure toward higher elevations.

First, the lowest site had a proportionally more efficient, but also hydraulically riskier, anatomical configuration. This was depicted by the significant differences in the maximum values of VA*_*std*_*, VA95*_*std*_*, DH*_*std*_*, KH*_*std*_* (higher), and VG*_*std*_* (smaller) at U1 compared with U3 occurring at the beginning of the ring (Figure 3B). Second, absolute values of VG were higher with increasing elevation enhancing the hydraulic safety of the individuals. Yet, this must be taken with care as the advantages and disadvantages of vessel grouping in relation to embolism are under debate (Carlquist, 2003; Martinez-Vilalta et al., 2012; Nardini et al., 2012; Scholz et al., 2013). Finally, the high number of correlations among anatomical traits at U3 compared with the other sites also supports the hypothesis of hydraulic limitations of the trees at this site (Oladi et al., 2014). This last result also showed how the anatomical structure of U3 trees is constrained to the point that their radial growth correlated with all the studied traits except the most plastic ones (VA and KH), resulting in VA*_*ind*_* being correlated with VA95*_*ind*_*.

The less safe and more efficient hydraulic system with decreasing elevation as inferred from anatomy could be related to higher water accessibility due to an earlier start of the growing season at lower elevations (Martínez del Castillo et al., 2016; Prislan et al., 2018) profiting from the more abundant precipitation in this period; the lower freezing-induced embolism probability during milder winters at the lower elevations (Sperry and Sullivan, 1992; Lemoine et al., 1999) and to deeper and more productive soils (Bouriaud et al., 2004) (observed during the sampling but not quantified).

### Increased Competition From Coppicing Cessation Might Lead to a More Conservative Wood Structure

Our results showed some indications to support our second hypothesis regarding the increase in hydraulic safety because of the land use change. After cessation of coppicing, coppices probably sprouted new shoots and kept the already growing stems, leading to the lower mean tree diameters as well as the high density and basal areas observed at U1 (Table 1). This might have triggered an increase in competition for water and evaporative demand.

Indeed, the different responses at U2 and U3 compared with U1 might be related to the heavier harvesting pressure at U1. The less plastic trait VA95 showed the highest number of differences between the periods before and after the land use change at U2 and U3 together with DH at U2 (Figure 4). Conversely, at U1, VA95 and DH were the only traits without a different intra-annual trend after cessation of coppicing. The increased competition agrees with the more conservative strategy observed at U1 as opposed to the previously observed increase in hydraulic conductance following competition release treatments (Lemoine et al., 2002; Diaconu et al., 2016; Noyer et al., 2017). VA_ind_, KH_ind_, and VD_ind_ at U1 turned as well toward a more conservative anatomical configuration after the land use change. However, with the present dataset, it is not possible to investigate in detail the anatomical responses to land use changes, as they are not acute but gradually developing over time and could be confounded with ontogenetic trends. This was the case for the differences observed in absolute values of the anatomical traits between the periods before and after the land use change that went in the same direction as the expected age-related trends.

### Intra-Ring Maximum Vessel Size Shows Different Climate Sensitivity but Little Differentiation to Extreme Years

The generally moderate associations of maximum vessel size (VA95) with climate are in line with the previous studies (Giagli et al., 2016; Rosell et al., 2017). However, climate sensitivity changed with elevation, with maximum vessel size being mainly limited by temperature at U1, precipitation at U2, and a mixture of temperature and precipitation at U3. Different sensitivities for the first formed vessels in tree rings (sectors 1–2) might be partly explained by the different drivers and processes involved in the formation of these vessels after cambial reactivation (Marchand et al., 2021). The negative correlation of first-sector VA95 with May temperature and the positive correlation with May precipitation at U1 likely points to reduced turgor because of the water limitation during cell enlargement (Arend and Fromm, 2007). For the later-formed vessels (sectors 3–5), it is difficult to directly link the negative associations with previous March and winter temperatures at U1 and U3, respectively, to tree physiology and cell formation processes. These negative associations probably represent memory effects in either trees or soil conditions (Zweifel et al., 2020; Walthert et al., 2021), as it might be also the case of the positive and negative associations with previous summer and winter temperatures, respectively, at U3. In contrast, the positive correlations of VA95 with summer precipitation at U2 and U3—remarkably the most striking correlation pattern found—is consistent with a reduction in seasonal drought stress and consequently increased turgor that might have triggered the formation of wider vessels (Hsiao, 1973). We cannot rule out the possibility that there are additional relevant relationships with VA95 and even more so with the other anatomical features that were not considered in these analyses if replication had been higher, which would have improved the robustness of the chronologies (EPS and Rbar). Similarly, daily climate records, if available, could strengthen and refine the understanding of the anatomical responses of the studied beech trees to climate.

Results were not conclusive regarding our third hypothesis. Extremely high and low summer temperatures induced only slight intra-annual differences in wood anatomy at U2 and U3, particularly in VA95*_*std*_* values (Figure 7). However, in addition to the inclusive definition of extreme years (10 of 37 years), the years with extremely high temperatures were clustered toward the end of the study period and the years with a low temperature toward the beginning. Therefore, as for the land use effects, our results could be confounded with ontogenetic trends. The same applies to the absolute values: differences are probably biased by the unbalanced timing of the considered extreme years throughout the study period. Conversely, extreme precipitation years were more scattered, but there were no differences between high- and low-summer precipitation years.

## Conclusion

In this study, we assessed the plasticity of different wood anatomical traits of European beech to elevation, coppicing cessation, and climate variability by investigating three beech stands along a 750 m elevational gradient in the Apennines (Italy). The anatomical structure between and within tree rings generally showed limited plasticity apart from species-specific and ontogenetic patterns. Yet, plasticity decreased and the hydraulic safety as inferred from the anatomical configuration increased toward the highest site. After land use change, trees at the most intensively exploited site kept a conservative strategy by maintaining their maximum vessel size in time. Furthermore, intra-ring maximum vessel size showed different climate sensitivity but little differentiation between normal and extreme years.

Our results show that several wood anatomical traits of beech are plastic at different time scales, from seasonal climate fluctuations to changes in stand dynamics as induced by changes in land use to long-term site conditions. The underlying external driver for this plasticity appears to be related to the water supply and water transport capacity in the wood, and also its efficiency and safety. Constraining hydrological conditions limit this plasticity and lead to closer coordination of the different anatomical traits and an anatomical configuration consistent with increased hydraulic safety. Nevertheless, the considered anatomical traits differed in both the absolute plasticity and immediacy of plasticity, with VA95 and DH being less plastic and VA, KH, RVA, VG, and VD being more plastic. However, it remains a challenge to clearly separate the externally induced medium- to longer-term responses from ontogenetically determined patterns, and insufficient replication could add to this uncertainty. This study, therefore, advocates for more comprehensive studies to further explore and verify the plasticity of wood anatomical traits in European beech, its extent and immediacy in response to short- to long-term environmental fluctuations to support sustainable forest ecosystems.

## Data Availability Statement

The raw data supporting the conclusions of this article will be made available by the authors, without undue reservation.

## Author Contributions

RT, BL, CCo, and CCa designed the study. CCa and CCo collected the samples and performed the field measurements. CCa and GA produced the anatomical data. JM with significant input from GA performed the statistical analysis and wrote the first draft of the manuscript. All authors contributed to manuscript revisions and approved the submitted version of the manuscript.

## Conflict of Interest

The authors declare that the research was conducted in the absence of any commercial or financial relationships that could be construed as a potential conflict of interest.

## Publisher’s Note

All claims expressed in this article are solely those of the authors and do not necessarily represent those of their affiliated organizations, or those of the publisher, the editors and the reviewers. Any product that may be evaluated in this article, or claim that may be made by its manufacturer, is not guaranteed or endorsed by the publisher.
