# Supplementary material for: Wood Anatomical Responses of European Beech to Elevation, Land Use Change, and Climate Variability in the Central Apennines, Italy
Source: Front Plant Sci. 2022 Mar 23;13:855741. doi: 10.3389/fpls.2022.855741 (PMC8983936; doi:10.3389/fpls.2022.855741)

Supplementary material

**Figure S1**: Time series of the mean absolute values of each of the anatomical traits at the different study sites: U1 – 1200 m a.s.l.; U2 – 1600 m a.s.l.; U3 – 1950 m a.s.l. The 5 extreme years with the highest/lowest temperatures (bottom dots) and precipitations (top dots) during the dry trimester June-July-August in the time period 1968-2004 are represented, as well as the sample depth (grey background). Vertical line indicates the year of the declaration of the National Park, when land use changed (1981). TRW: tree ring width, VA: vessel lumen area, VA95: 95th percentile of vessel lumen area, DH: mean hydraulic diameter, KH: theoretical hydraulic conductivity, VD: vessel density, RVA: proportion of accumulated vessel lumen to xylem area, and VG: vessel grouping index.


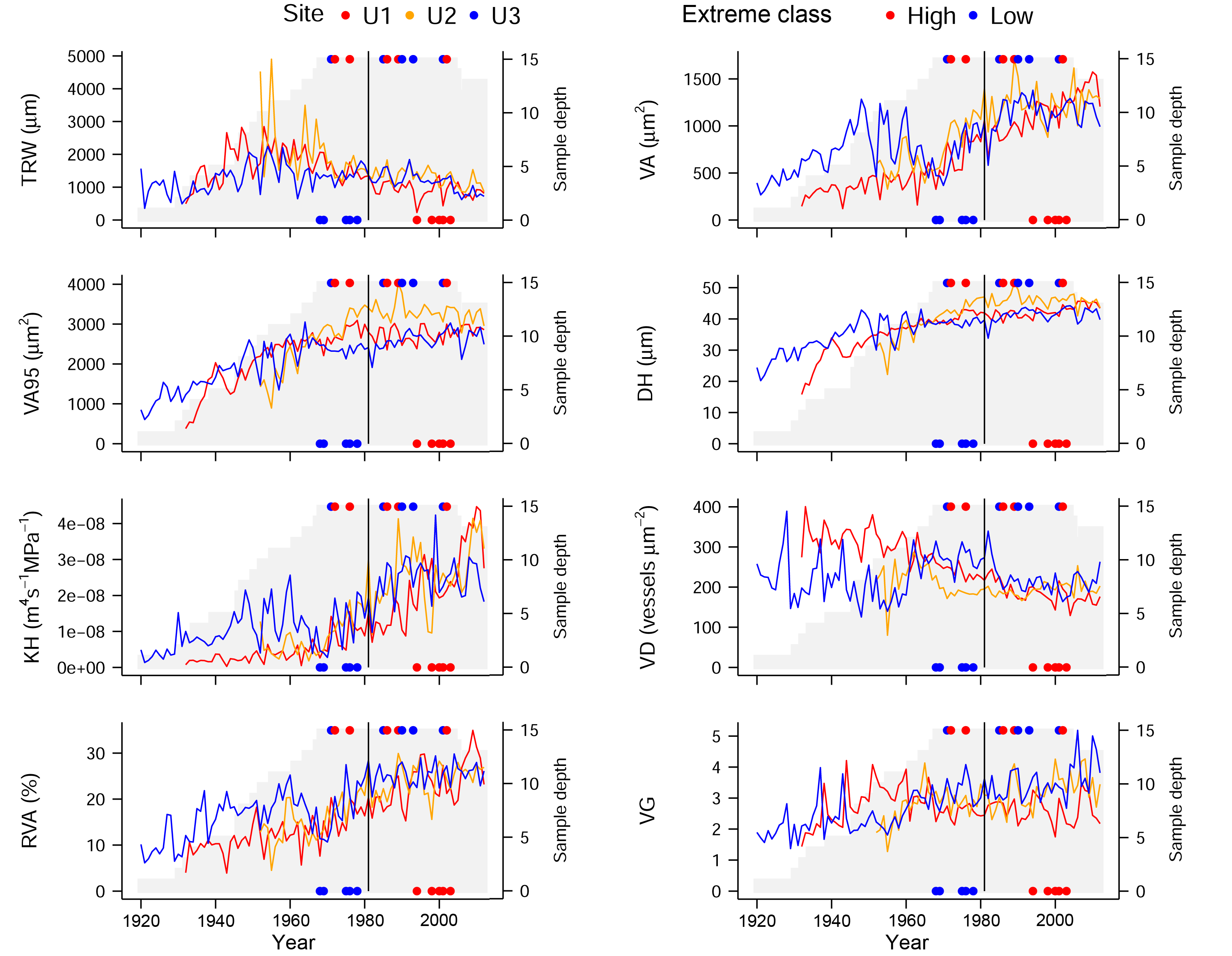


**Figure S2:** Tree ring width (TRW) values of each site (A), per land use period and site (B) and at the 5 years with highest/lowest temperatures during the dry trimester June-July-August (C) in the time period 1968-2004. Violin plots, with boxplots (1^st^, 2^nd^ and 3^rd^ quartiles), and mean values (dots); letters indicate significant differences between sites (permutation test of independence, P-value < 0.05). U1 – 1200 m a.s.l.; U2 – 1600 m a.s.l.; U3 – 1950 m a.s.l. Land use periods refer to the periods before (1968-1981) and after (1990-2004) the declaration of Ugni as Natural State Reserve in 1981 at the different study sites, which coincided with the cessation of coppicing.


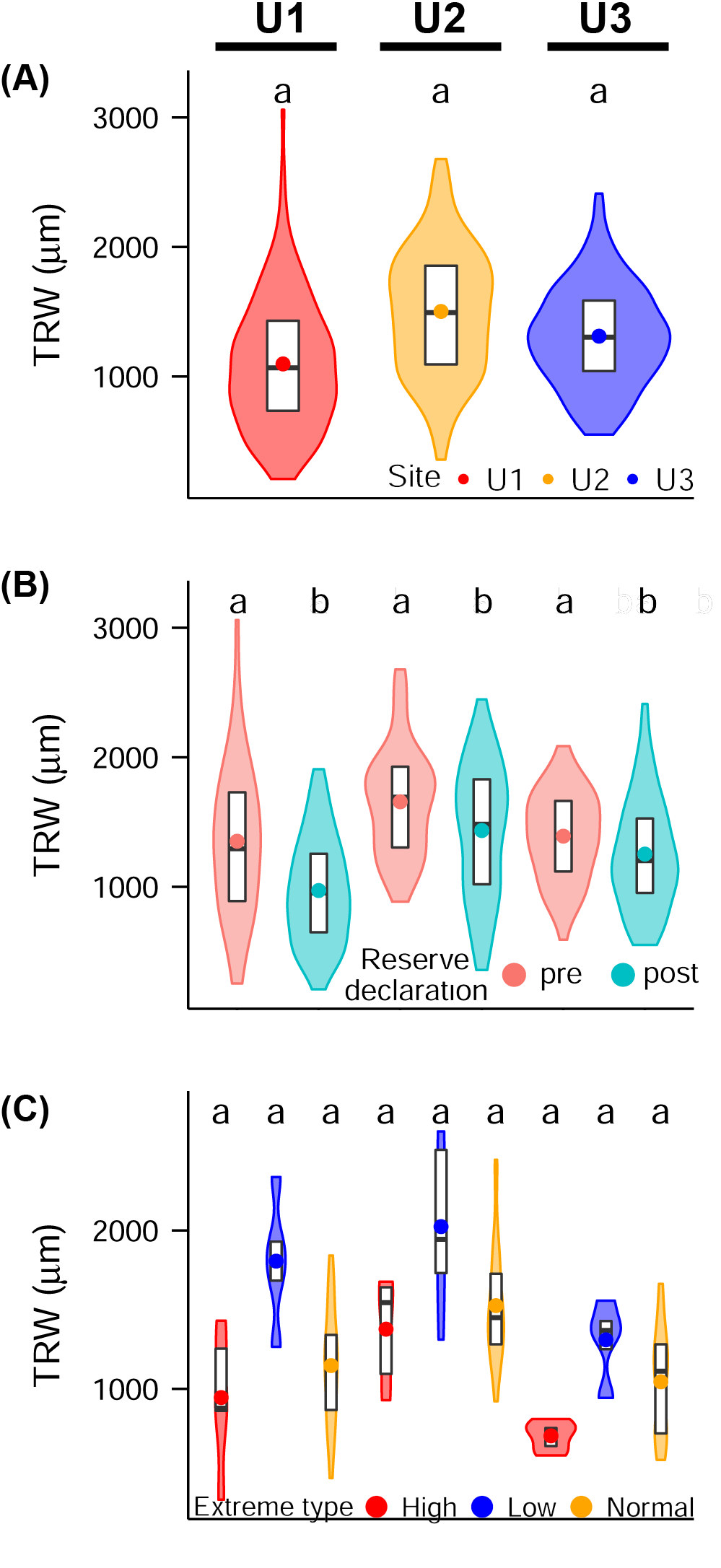


**Figure S3**: Inter-annual and standardized intra-annual values (standardized to the annual mean) of each of the anatomical traits studied at the periods before (1968-1981) and after (1990-2004) the declaration of Ugni as Natural State Reserve in 1981 at the different study sites, which coincided with abandonment of coppicing: U1 – 1200 m a.s.l.; U2 – 1600 m a.s.l.; U3 – 1950 m a.s.l. Left: Violin plots, with boxplots (1^st^, 2^nd^ and 3^rd^ quartiles), and mead values (dots); letters indicate significant differences between sites. Right: Mean values (dots) and 95% confidence interval (shaded area); letters indicate if values are significantly higher (**bold**), lower (*italics grey*). or if they are significantly different at all sites (regular format indicates the middle value). If both **bold** and *italics grey* are shown, this means that the mid-value was the only one that did not differ significantly. Permutation test of independence, P-value < 0.05. TRW: tree ring width, VA: vessel lumen area, VA95: 95th percentile of vessel lumen area, DH: mean hydraulic diameter, KH: theoretical hydraulic conductivity, VD: vessel density, RVA: proportion of accumulated vessel lumen to xylem area, and VG: vessel grouping index.


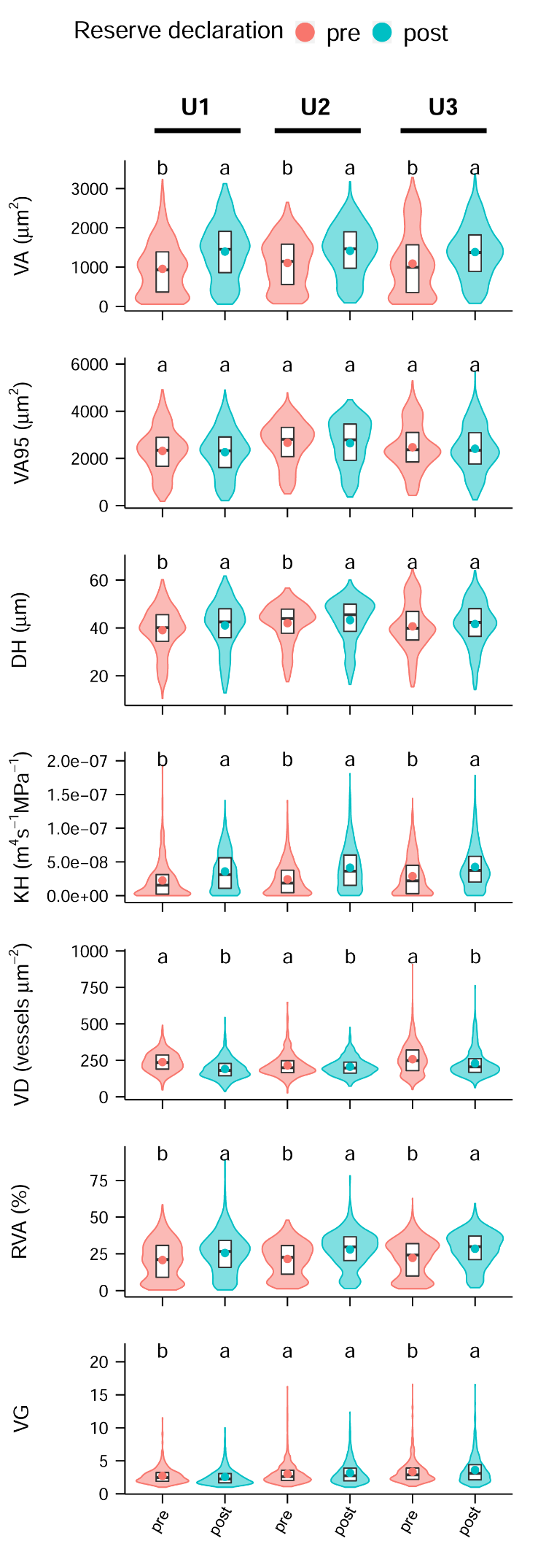

Supplement: Supplementary file 1 [file Data_Sheet_1.docx]
